# Prevalence and factors associated with carotid atherosclerosis in a Malagasy population with Type 2 diabetes mellitus: A cross‐sectional retrospective study

**DOI:** 10.1002/edm2.457

**Published:** 2023-10-10

**Authors:** Sitraka Angelo Raharinavalona, Rija Mikhaël Miandrisoa, Rija Eric Raherison, Thierry Razanamparany, Radonirina Lazasoa Andrianasolo, Andrianirina Dave Patrick Rakotomalala

**Affiliations:** ^1^ Cardiovascular Diseases and Internal Medicine departments Soavinandriana Hospital Center Antananarivo Madagascar; ^2^ Endocrinology Department Joseph Raseta Befelatanana University Hospital Center Antananarivo Madagascar

**Keywords:** cardiovascular risk factors, carotid atherosclerosis, carotid plaque, Type 2 diabetes

## Abstract

**Aim:**

Our study aims to determine the prevalence and factors associated with carotid atherosclerosis in Malagasy Type 2 diabetes mellitus (T2DM).

**Methods:**

This was a cross‐sectional retrospective study, carried out over a period of 30 months. The diagnosis of carotid atherosclerosis is established by the presence of a carotid plaque increased carotid intima‐media thickness ≥1.1 mm on Doppler ultrasound.

**Results:**

We included 132 T2DM. The prevalence of carotid atherosclerosis was 63.6% (38.6% carotid plaque and 25% intima‐media thickening). After univariate analysis, the factors associated with carotid atherosclerosis were age ≥70 years (3.28 [1.18–10, 62]), previous intake of oral antidiabetics (0.33 [0.14–0.73]), insulin (0.28 [0.11–0.66]) and angiotensin receptor blocker (0.45 [0.20–0.98]), and current smoking (5.93 [1.64–32.6]). After adjustment for age and gender, previous intake of oral antidiabetics (0.29 [0.13–0.64]), insulin (0.27 [0.12–0.61]) and angiotensin receptor blocker (0.40 [0.19–0.86]), and current smoking (5.98 [1.61–22.1]) were associated with carotid atherosclerosis.

**Conclusion:**

Smoking cessation, education on therapeutic compliance and comprehensive management of all cardiovascular risk factors and T2DM are therefore essential in order to reduce the occurrence of carotid atherosclerosis.

## Graphical abstract

Carotid atherosclerosis in patients with Type 2 diabetes mellitus was frequent and in the form of an atheroma plaque in the majority of cases. Given the associated factors, smoking cessation, education on therapeutic compliance and comprehensive management of all cardiovascular risk factors and control of diabetes are essential.

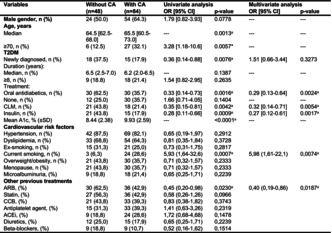

## 1 BACKGROUND

According to the World Health Organization (WHO), atherosclerosis is a variable combination of changes of the intima of in the intima of large and medium calibre arteries, consisting of a focal accumulation of lipids, complex carbohydrates, blood and blood products, fibrous tissue and calcium deposits, and associated with medial changes.^1^ It is the common cause of cardiovascular disease, dominated by ischemic heart disease and ischemic stroke.^2^ These are the leading causes of mortality and morbidity in Western countries and are on the increase in developing countries.^3^, ^4^, ^5^, ^6^ However, carotid atherosclerosis is a major and potentially preventable cause of ischemic stroke by carotid endarterectomy and stenting.^7^

In addition, diabetes mellitus is also a real public health problem. According to the International Diabetes Federation (IDF), the exponential increase in its frequency will be mainly observed in Sub‐Saharan Africa, around 134%.^8^ It is associated with two to four times higher cardiovascular mortality.^9^ Indeed, it constitutes an independent risk factor for the accelerated development of atherosclerosis, including carotid. Among the known pathological mechanisms linking diabetes and atherosclerosis are dyslipidemia, hyperglycemia with advanced glycation end products, increased oxidative stress and inflammation.^10^ The prevalence of carotid atherosclerosis in diabetes could be up to 73%.^11^

In the absence of data on the extent of this pathology among the Malagasy diabetic population, we conducted this study to determine the prevalence and factors associated with carotid atherosclerosis in Type 2 diabetics (T2DM) seen at the Soavinandriana Hospital Center, located in the capital of Madagascar, Antananarivo.

## 2 MATERIALS AND METHODS

### 2.1 Study design and patients

The study was carried out in the cardiovascular diseases and Internal Medicine departments of the Soavinandriana Hospital Center, Antananarivo, capital of Madagascar. These are reference departments for the management of cardiovascular, metabolic and endocrine diseases at the Hospital.

This was an analytical cross‐sectional retrospective study, conducted over a period of 2 years and 6 months from 1 January 2020 to 30 June 2022. The study population consists of patients with T2DM seen in its departments. We included all known or newly diagnosed T2DM, benefited from a vascular Doppler ultrasound of the supra‐aortic trunks and presenting or not carotid atherosclerosis. We excluded from this study patients with incomplete medical records.

### 2.2 Determination of T2DM

Diabetes mellitus was diagnosed with fasting plasma glucose ≥7 mmol/L (126 mg/dL); glycated haemoglobin ≥6.5% (48 mmol/mol) or random plasma glucose ≥11.1 mmol/L (200 mg/dL) associated with classic symptoms of hyperglycemia.^12^ Type 2 typing was retained in view of the patient's age (>35 years), overweight/obesity, family history of T2DM and the absence of obvious secondary causes (chronic pancreatitis, endocrinopathies, long‐term glucocorticoid use).

### 2.3 Determination of carotid atherosclerosis

The search for carotid atherosclerosis was done using a Mindray Resona I9® brand vascular doppler ultrasound with a high frequency linear probe (7.5 MHz). In order to measure the carotid intima‐media thickness (CIMT), longitudinal sections in 2D mode of the right and left common carotid arteries were taken 1–2 cm upstream of the carotid bifurcations.

Carotid plaque was defined as a focal structure encroaching into the arterial lumen or focal thickening of the surrounding wall >50% or CIMT >1.5 mm.^13^ Carotid atherosclerosis has been defined as the presence of carotid plaque or diffuse thickening of the carotid wall (mean CIMT ≥1.1 mm).^14^, ^15^ The results were classified into three Strandness categories: normal (no atherosclerotic lesions), stenosis <50% and stenosis ≥50%.^16^, ^17^

### 2.4 Clinical and laboratory data

The parameters studied were demographic data (gender, age); T2DM (duration, previous antidiabetic treatment, glycated haemoglobin); the associated cardiovascular risk factors (hypertension, smoking, dyslipidemia, menopause, microalbuminuria, overweight/obesity); other previous treatments (antihypertensives, antiplatelet agent, statins) and the results of carotid arterial Doppler (presence of atherosclerosis, EIMC, stenosis <50% and stenosis ≥50%).

The glycated haemoglobin test was performed using a High‐Performance Liquid Chromatography method. Diabetes was said to be controlled if the glycated haemoglobin was less than 7% (<53 mmol/mol). Hypertension was defined as systolic/diastolic blood pressure ≥ 140/90 mmHg. The colorimetric technique on Alinity C from Abbott 8501 was used for the assays of the lipid parameters. And patients with low‐density lipoprotein cholesterol (LDL‐C) outside the European Society of Cardiology targets^18^ or taking a lipid‐lowering drug were considered to have dyslipidemia.

### 2.5 Statistical analysis

The data was collected from a pre‐established collection grid, from patient files. They were used by Epi Info™ software version 3.5.4. (United States Centers for Disease Control and Prevention in Atlanta, Georgia). The qualitative and quantitative variables are expressed respectively as a percentage and as an average with standard deviation. A univariate statistical analysis was performed according to the presence of carotid atherosclerosis using the odds ratio with 95% confidence interval and statistical significance *p* < .05. Then, the odds ratio was adjusted according to age and gender.

## 3 RESULTS

During the study period, there were 141 T2DM patients who underwent vascular ultrasound of the supra‐aortic trunks. One hundred and thirty‐two patients were included in the study who met the eligibility criteria and nine were excluded. The prevalence of carotid atherosclerosis was 63.6%, of which 38.6% was in the form of carotid plaque and 25% intima‐media thickening. Mean CIMT was 2.12 ± 1.63 mm. Carotid stenosis was <50% and ≥50% in 52.3% and 11.3% of cases, respectively.

Table 1 presents the general characteristics of the study population. Mean age of patients was 64.7 ± 8.4 years with extremes of 38 and 82 years. Mean duration of diabetes was 6.2 ± 5.4 years. Oral antidiabetics and insulin were previously prescribed in 45.5% and 27.3% of cases, respectively. Note that a patient could have one or more antidiabetics. Mean glycated haemoglobin was 9.3% ± 2.5% with extremes of 6.0% and 16.2%. The main associated cardiovascular risk factors were hypertension (84.1%) and dyslipidemia (65.9%).

**TABLE 1:** | General characteristics of the study population (*N* = 132).

Variables	Numbers	Percentage
Gender		
Male	78	61.8
Female	54	38.2
Age (years)		
≤49	9	6.8
[50–59]	18	13.6
[60–69]	72	54.6
≥70	33	25.0
Type 2 diabetes mellitus		
Newly diagnosed	40	30.2
Duration (years)		
[1–5]	48	36.4
[6–10]	32	24.3
≥11	12	9.1
Treatment		
Oral antidiabetics	60	45.5
None	42	31.8
Comprehensive lifestyle modification	39	29.5
Insulin	36	27.3
Glycated haemoglobin <7%	39	29.5
Cardiovascular risk factors		
Hypertension	111	84.1
Dyslipidemia	87	65.9
Ex‐smoking	36	27.3
Current smoking	27	20.5
Overweight/obesity	51	38.6
Menopause	51	38.6
Microalbuminuria	27	20.5
Other previous treatments		
Angiotensin receptor blockers	66	50.0
Statin	63	47.7
Calcium‐channel blockers	54	40.9
Antiplatelet agent	48	36.4
Angiotensin‐converting enzyme inhibitor	33	25.0
Diuretics	27	20.5
Beta‐blockers	18	13.6

Table 2 presents factors associated with carotid atherosclerosis. In univariate analysis, the factors associated with carotid atherosclerosis were the age ≥70 years (OR 3.28 [1,18–10.62]), T2DM newly diagnosed (OR 0.36 [0.14–0.88]), previous use of oral antidiabetics (OR 0.33 [0.14–0.73]) and insulin (OR 0.28 [0.11–0.66]).

**TABLE 2:** Factors associated with carotid atherosclerosis (*N* = 132).

Variables	Without CA	With CA	Univariate analysis		Multivariate analysis	
	(*n* = 48)	(*n* = 84)	OR (95% CI)	*p*‐value	OR (95% CI)	*p*‐value
Male gender, *n* (%)	24 (50.0)	54 (64.3)	1.79 (0.82–3.93)	.0778	–	–
Age, years						
Median	64.5 [62.5–68.0]	65.5 [60.5–73.0]	–	.0013*	–	–
≥70, *n* (%)	6 (12.5)	27 (32.1)	3.28 (1.18–10.6)	.0057*	–	–
T2DM						
Newly diagnosed, *n* (%)	18 (37.5)	15 (17.9)	0.36 (0.14–0.88)	.0076*	1.51 (0.66–3.44)	.3273
Duration (years)						
Median, *n* (%)	6.5 (2.5–7.0)	6.2 (2.0–6.5)	–	.1387	–	–
≥8, *n* (%)	9 (18.8)	18 (21.4)	1.54 (0.82–2.95)	.2635		
Treatment						
Oral antidiabetics, *n* (%)	30 (62.5)	30 (35.7)	0.33 (0.14–0.73)	.0016*	0.29 (0.13–0.64)	.0024*
None, *n* (%)	12 (25.0)	30 (35.7)	1.66 (0.71–4.05)	.1404	–	–
CLM, *n* (%)	21 (43.8)	18 (21.4)	0.35 (0.15–0.81)	.0042*	0.32 (0.14–0.71)	.0054*
Insulin, *n* (%)	21 (43.8)	15 (17.9)	0.28 (0.11–0.66)	.0009*	0.27 (0.12–0.61)	.0017*
Mean A1c, % (±SD)	8.44 (2.38)	9.93 (2.59)	–	<.0001*	–	–
Cardiovascular risk factors						
Hypertension, *n* (%)	42 (87.5)	69 (82.1)	0.65 (0.19–1.97)	.2912		
Dyslipidemia, *n* (%)	33 (68.8)	54 (64.3)	0.81 (0.35–1.84)	.3728		
Ex‐smoking, *n* (%)	15 (31.3)	21 (25.0)	0.73 (0.31–1.75)	.2817		
Current smoking, *n* (%)	3 (6.3)	24 (28.6)	5.93 (1.64–32.6)	.0007*	5.98 (1.61–22.1)	.0074*
Overweight/obesity, *n* (%)	21 (43.8)	30 (35.7)	0.71 (0.32–1.57)	.2333		
Menopause, *n* (%)	21 (43.8)	30 (35.7)	0.71 (0.32–1.57)	.2333		
Microalbuminuria, (%)	9 (18.8)	18 (21.4)	0.65 (0.25–1.71)	.2239		
Other previous treatments						
ARB, (%)	30 (62.5)	36 (42.9)	0.45 (0.20–0.98)	.0230*	0.40 (0.19–0.86)	.0187*
Statin, (%)	27 (56.3)	36 (42.9)	0.58 (0.26–1.26)	.0966		
CCB, (%)	21 (43.8)	33 (39.3)	0.83 (0.38–1.82)	.3743		
Antiplatelet agent, (%)	15 (31.3)	33 (39.3)	1.41 (0.63–3.26)	.2319		
ACEi, (%)	9 (18.8)	24 (28.6)	1.72 (0.68–4.68)	.1478		
Diuretics, (%)	12 (25.0)	15 (17.9)	0.65 (0.25–1.71)	.2239		
Beta‐blockers, (%)	9 (18.8)	9 (10.7)	0.52 (0.16–1.62)	.1514		

Abbreviations: ACEi, Angiotensin‐converting enzyme inhibitor; ARB, Angiotensin receptor blockers; CA, carotid atherosclerosis; CI, Calcium‐channel blockers CI, Confidence Interval; CLM, Comprehensive lifestyle modification; A1c, Glycated haemoglobin; OR, Odds ratio. T2DM, Type 2 diabetes mellitus.

* *p*‐value <.05.

After adjustment according to age and gender, previous use of oral antidiabetics and insulin were protective factors for carotid atherosclerosis. Current smoking and previous use of angiotensin receptor blockers were significantly associated with carotid atherosclerosis, with adjusted ORs of 5.98 [1.61–22.1] and 0.40 [0.19–0.86], respectively.

## 4 DISCUSSION

In the present study, the prevalence of carotid atherosclerosis was 63.6%. Carotid stenosis was ≥50% in 11.4% of cases.

This was superimposable on the findings of French studies in which 65.4% of a diabetic population presented with carotid atherosclerosis and the proportion of stenoses <50% and ≥50% were 64.6% and 4.1%, respectively.^17^, ^19^ However, in other Chinese and Korean studies found lower prevalence ranging from 30.8% to 45%.^20^, ^21^, ^22^ In the study by Yang et al., 82.25% of patients had ≤49% stenosis.^23^ These differences could be linked to the disparity of the population studied, the diagnostic criteria for carotid atherosclerosis used in each study and the operator's dependence on ultrasound. Nevertheless, carotid atherosclerosis remains common in patients with T2DM. Determining the degree of stenosis is always essential for subsequent medical and/or surgical management.

Male predominance in the present study was also found by other authors.^17^, ^19^, ^20^ In a study conducted by Herinirina et al. in a general Malagasy population, male gender was a determining factor in the increase in common carotid intima‐media thickness (*p* value = .008).^24^ Also, according to a literature review conducted by Man, et al. male subjects develop earlier, more inflamed and unstable atherosclerotic plaques.^25^ Thus, in a South Korean study, 55.1% of males had carotid atherosclerosis versus 49.6% of females with a significant *p* value <.001.^14^

Advanced age was a factor in the occurrence of carotid atherosclerosis in the present study as in other studies.^20^, ^22^ According to Tyrrell and Goldstein, aging is associated with a decline in mitochondrial function and an increase in interleukin–6 levels in the vascular system, and these two effects probably accelerate atherosclerosis independently of chronic hyperlipidemia.^26^ Duration of diabetes was not significantly different between the two groups of our patients. However, Vouillarmet et al.^19^ as well as Kawamori et al.^27^ had established that long duration of diabetes constituted a risk factor for carotid atherosclerosis. Systematic screening for carotid atherosclerosis in T2DM should be done as soon as diabetes is diagnosed, since this complication was the first of the diabetic disease in about a third of cases.^21^

In the present study, the adequate management of diabetes by comprehensive lifestyle modification, oral antidiabetics and insulin, was a protector of carotid atherosclerosis. According to the review by Ishii, certain oral antidiabetics like metformin, α‐glucosidase inhibitors, pioglitazone, dipeptidyl peptidase‐4 inhibitors reduce carotid intima‐media thickness.^28^ Indeed, it promotes the achievement of a glycemic control. And increased glycated haemoglobin was a factor associated with carotid intima‐media thickening and carotid atherosclerosis, as Ling et al. also found.^29^ Decrease antioxidant activity associated with diabetes mellitus is also implicated in the development of atherosclerosis.^30^

Even if no significant association was not objectified in the present study, hypertension and dyslipidemia remain the main cardiovascular risk factors most associated with diabetes as in the literature.^29^ Moreover, other authors had shown that these risk factors were significantly associated with carotid atherosclerosis.^20^, ^31^ Since, these are pathological conditions that aggravate the development and progression of atherosclerotic lesions.^32^ The present study and that of Hu et al.^31^ confirmed the harmful effect of current smoking in the onset of carotid atherosclerosis, independently of other factors. Thus, smoking cessation and early and adequate management of other modifiable risk factors are essential.

The present study confirmed the property of the angiotensin receptor blockers on cardiovascular protection. Other authors have also objectified significant associations between the use of antihypertensive and antiplatelet agents, and the presence of carotid atherosclerosis.^20^ In addition, the proportions of patients on antihypertensive and lipid‐lowering medication were lower than those for hypertension and dyslipidemia. What was also observed in the study by Golan et al.^33^ All of this implies that either the patients were non‐compliant with the treatment, or they had not consulted a practitioner to receive the drug prescription, or the practitioner had not followed the recommendations. Patient education on therapeutic compliance and the promotion of continuing medical education are still essential to sustain.

### 4.1 Limitations of the study

The present study was limited by the size of the sample, not allowing its extrapolation to the entire general population in Madagascar. Its retrospective nature also does not allow the evaluation of certain important information that did not appear in the medical records, such as other cardiovascular risk factors and data on surgical management.

## 5 CONCLUSION

During T2DM, carotid atherosclerosis was frequent and in the form of an atheroma plaque in the majority of cases. Given the associated factors, smoking cessation, education on therapeutic compliance and comprehensive management of all cardiovascular risk factors and control of diabetes are essential. This care must be multidisciplinary. Given the limitations of the study, carrying out a multicentre study with a large population, even retrospective, will already confirm the factors associated with carotid atherosclerosis and identify other factors. A prospective study is also interesting to determine the progression of carotid lesions and the occurrence of long‐term cardiovascular complications.

## AUTHOR CONTRIBUTIONS

**Sitraka Angelo Raharinavalona:** Conceptualization (equal); data curation (equal); formal analysis (equal); investigation (equal); methodology (equal); software (equal); validation (equal); visualization (equal); writing – original draft (equal); writing – review and editing (equal). **Rija Mikhaël Miandrisoa:** Validation (equal); visualization (equal). **Rija Eric Raherison:** Validation (equal); visualization (equal). **Thierry Razanamparany:** Visualization (equal). **Radonirina Lazasoa Andrianasolo:** Supervision (equal); validation (equal); visualization (equal); writing – review and editing (equal). **Andrianirina Dave Patrick Rakotomalala:** Supervision (equal); validation (equal); visualization (equal); writing – review and editing (equal).

## FUNDING INFORMATION

The current study was not supported by any funding source.

## CONFLICT OF INTEREST STATEMENT

The authors have declared that they have no competing interest.

## Data Availability

The data sets used and/or analysed during the current study are available from the corresponding author on reasonable request.
